# Six complete genome sequences of a putative nitrogen-fixing bacteria from the genus *Agarivorans* sp. isolated from the seagrass, Zostera marina

**DOI:** 10.1128/mra.00577-25

**Published:** 2025-09-25

**Authors:** Jackie Badillo, Diane Brache-Smith, E. Maggie Sogin

**Affiliations:** 1 Molecular Cell Biology, School of Natural Sciences, University of California 118577 , Merced, USA; California State University San Marcos, San Marcos, California, USA

**Keywords:** seagrass, agarivorans, nitrogen fixation

## Abstract

We report six complete genome sequences of *Agarivorans* sp. isolated from *Zostera marin*a’s root endosphere and rhizoplane. The genomes will facilitate future studies investigating the role of these putative nitrogen-fixing bacteria in supporting seagrass growth and metabolism.

## ANNOUNCEMENT

The seagrass, *Zostera marina*, occurs throughout coastal regions in the Northern Hemisphere and contributes to marine carbon sequestration (1). The microbial communities associated with *Z. marina* are thought to be crucial for supporting seagrass ecosystems and carbon burial. Here, we report on complete genome sequences of six strains of *Agarivorans* sp. isolated from *Z. marina’s* rhizoplane and endosphere. *Agarivorans* sp. is known to degrade complex polysaccharides and is predicted to play a role in nutrient cycling (2). These genomes will enable investigations into the potential role of *Agarivorans* sp. in biogeochemical cycling in *Z. marina* meadows.

Individual *Z. marina* plants were collected from Bodega Bay, CA (38.333446°N, 123.058785°W). The adhering sediments were removed from the roots. After which, we collected the rhizoplane inoculum by sonicating the roots in 10 mL of sterile filtered artificial seawater. The roots were then macerated and sonicated to collect inoculum from the endosphere. The slurries were serially diluted in sterile artificial seawater and plated onto agar plates supplemented with 5% v/v seagrass metabolite extract. Plates were incubated at 14°C for 30 days, after which single colonies were picked and subcultured three times on marine broth (MilliporeSigma) plates to ensure purity. Overnight cultures in marine broth were prepared prior to DNA extraction using Quick-DNA Miniprep Plus Kit (ZymoResearch). Extracted DNA was sent to Plasmidsaurus (Eugene, OR) for Nanopore sequencing using the ONT Ligation sequencing kit v.14 (SQK-LSK114) library preparation kit without size selection or fragmentation according to the manufacturer’s instructions. The library was sequenced on a PromethION 24 device using R10.4.1 flow cell chemistry.

Default parameters were used for all software described in this study unless otherwise specified. Base calling was completed using Guppy (v.6.1.5). Quality control was conducted with Filtlong (v.0.2.1) using the flags –min length 1000 –keep percent 90 to remove reads shorter than 1,000 bp and retain the top 90% of reads based on quality (3). Autocycler (v.0.2.1) (4) generated a consensus *de novo* assembly using CANU (v.2.4) (5), flye (v.2.9.5) (6), miniasm (v.0.3)(7), necat (v.0.0.1)(8), nextdenovo (v.2.5.2) (9), and raven (v.1.8.3) (10) and reported that all six genomes assembled into a single, circular contig. The circular assembly graph was manually inspected and subsequently reoriented to the origin of replication using Dnaapler (v.1.2.0) (11).

**Fig 1:**
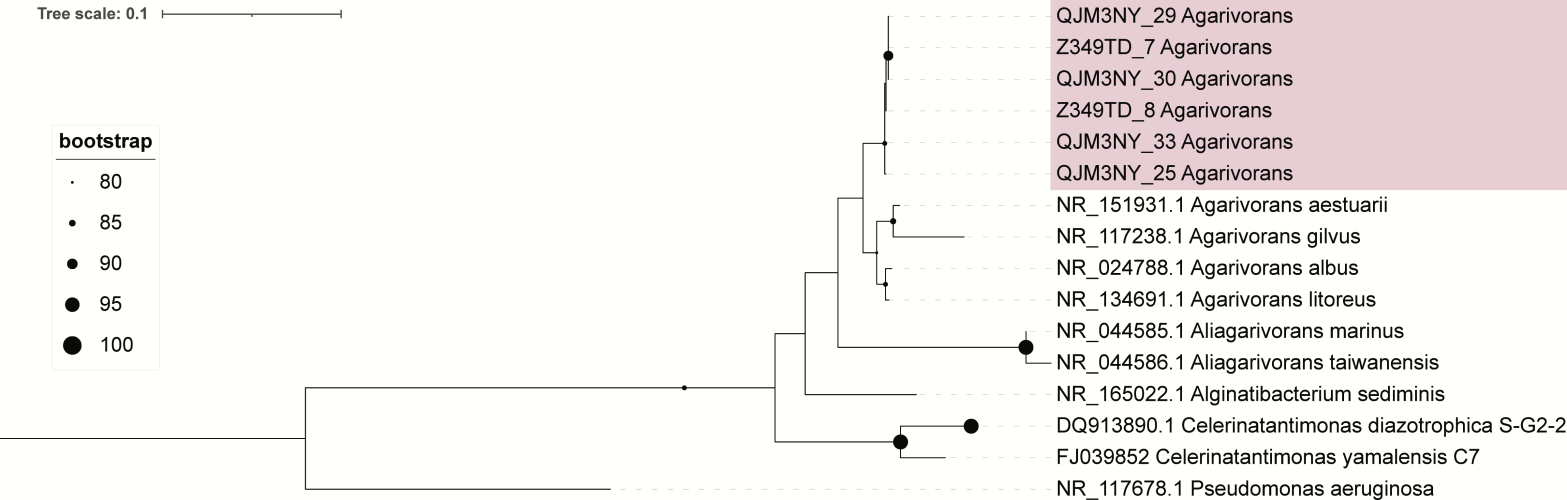
16S rRNA phylogenetic tree groups the isolates within the *Agarivorans* genus. The tree was calculated using extracted 16S rRNA sequences from each genome and NCBI sequences with >90% similarity to the query sequence. The tree was built from an MAFFT alignment in IQ-TREE (17, 18).

Genome analysis was conducted in Kbase (12). Using Quast (v.4.4), genome size was estimated to be between 4.99 and 5.08 Mb, average G+C content 44.8%, and an average of 106-fold genome coverage (Table 1) (13). The lineage_wf pipeline in CheckM (v.1.0.18) estimated each genome to be 100% complete with <1.2% contamination (14). GTDB-Tk (v.2.3.2) classified all isolates within the *Agarivorans* genus, with average nucleotide identity (ANI) values between 79.99 and 80.41% to the closest reference genome (GenBank GCF023238625.1) (15). A phylogenetic tree on the 16S rRNA gene confirmed that the isolates belonged to the *Agarivorans* genus (Fig. 1). Each genome was annotated using NCBI’s PGAP pipeline (v.6.10)(16), which resulted in 4,432–4,496 coding domain sequences per genome. All six genomes contained the genes needed for nitrogen fixation (i.e., *nifHDK*).

**TABLE 1:** Summary of complete genome sequences generated in this study

Isolate ID	Nr Reads	rN50	ANI %	Genome size (mbp)	GC Content (%)	Genome Coverage	Completeness (%)	Contamination (%)	Nr. Genes	RNA	CDS
Z349TD_7	97,423	10,936	80	5.06	44.8	75.7	100	1.17	4614	118	4496
QJM3NY_30	60,470	16,748	80	5.06	44.8	66.7	100	1.17	4613	118	4495
QJM3NY_29	171,197	9,539	80.4	5.06	44.8	119.5	100	1.17	4612	118	4494
QJM3NY_33	169,323	11,428	79.9	5.08	44.97	178.6	100	0.99	4605	118	4487
QJM3NY_25	83,619	9,484	80	4.99	44.98	68.2	100	0.99	4578	119	4459
Z349TD_8	115,399	12,086	79.9	5.03	44.91	129.4	100	0.99	4550	118	4432

## Data Availability

All raw data from this project has been deposited into NCBI under project id PRJNA1260012. The genome assemblies are available in GenBank under the accession numbers CP194036.2, CP194037.2, CP194038.2, CP194039.2, CP194040.2, CP194041.2. Raw sequencing reads are available on NCBI’s Short Read Archive (SRA) under the accession numbers SRX28988440, SRX28988439, SRX28988438, SRX28988437, SRX28988436 and SRX28988435.
